# Preparative Biocatalytic Synthesis of α-Ketoglutaramate

**DOI:** 10.3390/ijms222312748

**Published:** 2021-11-25

**Authors:** Maksim Nikulin, Viktor Drobot, Vytas Švedas, Boris F. Krasnikov

**Affiliations:** 1 Belozersky Institute of Physicochemical Biology, Lomonosov Moscow State University, Lenin Hills 1, Bldg. 40, 119991 Moscow, Russia; nikulin000@mail.ru (M.N.); linux776@gmail.com (V.D.); 2 Faculty of Bioengineering and Bioinformatics, Lomonosov Moscow State University, Lenin Hills 1, Bldg. 73, 119991 Moscow, Russia; 3 Centre for Strategic Planning of FMBA of the Russian Federation, Pogodinskaya St., Bld.10, 119121 Moscow, Russia

**Keywords:** α-ketoglutaramate, L-amino acid oxidase, preparative biocatalytic synthesis, ω-amidase, substrate

## Abstract

α-Ketoglutaramate (KGM) is an underexamined metabolite of L-glutamine in the metabolic pathway of glutaminase II of α-ketoglutarate formation. Presumably, KGM may be a biomarker of hepatic encephalopathy and other hyperammonemic diseases. This metabolite is a substrate for the ω-amidase enzyme and is used to determine its activity in the study of the biochemistry of various types of cancer. However, the commercial unavailability of KGM hinders its widespread use. Methods for the preparative synthesis of KGM are known, but they either do not provide the proper yield or proper purity of the target product. In this work, a detailed description of the procedures is given that allows the production of KGM with a purity above 97% and a yield of the target product above 75% using L-amino acid oxidase from *C. adamanteus* as a catalyst of L-glutamine conversion. KGM can be obtained both in the form of a highly concentrated aqueous solution and in the form of crystals of sodium salt. The developed methods can be used both for scaling up the synthesis of KGM and for creating economical biocatalytic technologies for the production of other highly purified preparations.

## 1. Introduction

As the most abundant amino acid in the human body, L-glutamine is involved in many biochemical transformations. The cells of most cancers reprogram their metabolism to a greater extent to α-ketoglutarate, a Krebs cycle metabolite formed from L-glutamine, to produce high-energy succinyl-CoA through oxidative decarboxylation catalyzed by α-ketoglutarate dehydrogenase complex [1,2]. Cancer cells also use NADPH-dependent pathways for carboxylation of α-ketoglutarate with the formation of isocitrate, which is a substrate for the production of nucleic acids, lipids, and other building blocks necessary for the rapid proliferation. α-Ketoglutarate used in these processes is formed from L-glutamine in two independent pathways: the glutaminase I pathway, where the intermediate metabolite is L-glutamate, and the glutaminase II pathway, in which the key metabolite is α-ketoglutaramate (KGM) [3]. The low efficiency of chemotherapeutic agents from the group of inhibitors of the glutaminase I pathway may possibly be due to the use by cancer cells of an alternative glutaminase II pathway for the utilization of L-glutamine [4]. Therefore, selective inhibitors of glutaminase II pathway enzymes could be of interest as new chemotherapeutic drugs. The key metabolite of the glutaminase II pathway is KGM, which is converted to α-ketoglutarate by the hydrolysis of the amide group catalyzed by ω-amidase—an underappreciated, but important enzyme in L-glutamine metabolism [5,6].

ω-Amidase is widely distributed in nature and is present in relatively high concentrations in all mammalian tissues. ω-Amidase demonstrates an ambiguous role in cancer biochemistry: on the one hand, inhibition of ω-amidase in colon cancer cells causes cell cycle arrest, on the other hand, overexpression of ω-amidase in HeLa cells also inhibits the cell cycle [7,8]. The study of the role of ω-amidase in cancer biology continues, and to control the activity of this enzyme, it is necessary to have high-purity KGM, which is a natural substrate of ω-amidase. In addition, it is suggested that KGM may be a biomarker of hepatic encephalopathy and other hyperammonemic diseases [9,10,11]. Thus, the development of methods for the synthesis of high-purity KGM is currently necessary to ensure the ongoing biochemical research.

In 1953, Meister first developed a procedure for obtaining KGM from L-glutamine using enzymes [12]. For the oxidative deamination of L-glutamine, *C. adamanteus* snake venom, containing a significant amount of L-amino acid oxidase, was used. The synthesis was carried out in an aqueous solution in the presence of catalase, which was applied to remove the hydrogen peroxide formed during the reaction. Upon reaching the degree of conversion of 95%, the protein was removed from the reaction mixture and the resulting solution was passed through a chromatographic column with a cation-exchange resin in the H^+^-form. The collected eluate was further decolorized by treatment with activated charcoal and neutralized with a solution of barium hydroxide (barite water). KGM was concentrated in the form of barium salt, precipitated with ethanol, and recrystallized. This protocol was modified in 2009 for the analysis of ω-amidase activity during the isolation and purification of the ω-amidase enzyme [13]. After purification on a cation exchange column and decolorization with activated charcoal, KGM solution was neutralized with 1 M sodium hydroxide to pH 6.0. The yield of KGM, according to this modified protocol, was 58%, but the resulting preparation was contaminated with a significant amount of impurities in relation to KGM: about 5% of 5-oxoproline (pyroglutamic acid), and about 1% of α-ketoglutarate [13].

In 2020, Shen et al. proposed a three-step organic synthesis of KGM [14]. Using L-2-hydroxyglutaramate as a starting material, the authors synthesized α-ketoglutaramic acid, including: (I) the introduction of a protective group on the carboxyl, (II) the oxidation of the hydroxyl group using Dess–Martin Periodinane, and (III) the removal of protective group. The authors report a 53% yield of the product, and obtaining α-ketoglutaramic acid in the form of opaque brown crystals. In this work, the structure of the target compound was proved by the NMR method; however, the authors do not provide data on its chemical purity.

This work presents an optimized one-step synthesis of α-ketoglutaramate from L-glutamine, catalyzed by L-amino acid oxidase from *C. adamanteus*, which made it possible to obtain a KGM preparation with high purity (more than 97%) and a significant yield (more than 75%).

## 2. Results and Discussion

**Figure 1:**
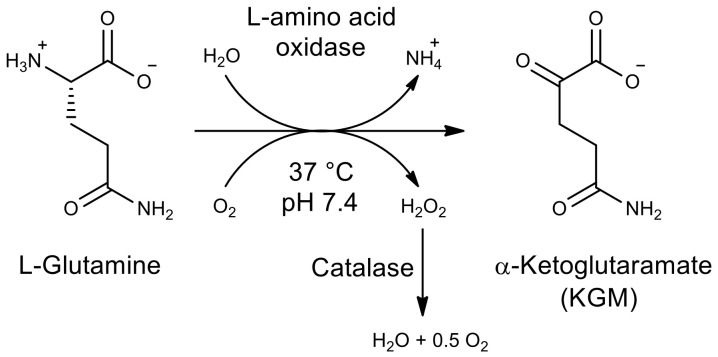
Scheme of biocatalytic synthesis of α-ketoglutaramate (KGM).

**Figure 2:**
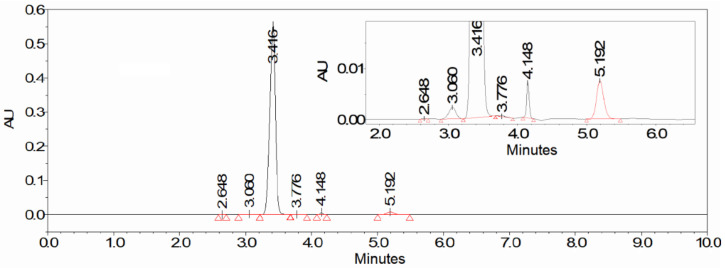
Chromatogram of the synthesized α-ketoglutaramate (HPLC-system: Waters 1525 Binary HPLC Pump, Waters 2489 UV/Visible Detector, Waters 2707 Autosampler; analysis conditions are specified in Section 3.3). Retention times: L-glutamine—2.65 min (≤0.05%), α-ketoglutaramate—3.42 min (≥97%), α-ketoglutarate—3.78 min (~0.03%), 5-oxoproline—5.19 min (~1.2%). Other peaks are unidentified impurities (≤1.5%).

**Figure 3:**
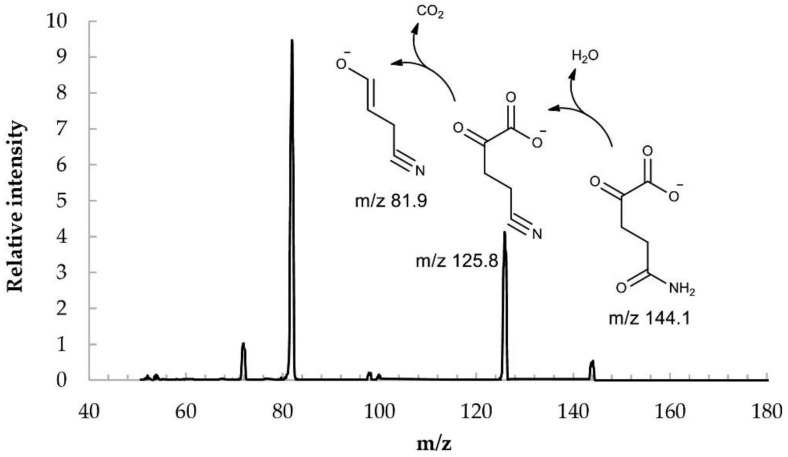
MS/MS spectrum of the synthesized preparation of α-ketoglutaramate (3200 Q TRAP LC/MS/MS System, Applied Biosystems, triple quadrupole). Concentration of KGM was 10 μM. Solvent—acetonitrile (LC/MS)/water MQ (1:1), direct infusion. Electrospray ionization (ESI-MS), ionization temperature 400 °C. Detection mode—negative ions. IonSpray Voltage 4150 V, declustering potential 15 V, entrance potential 4 V, collision energy 20 eV. Ion peak identification: 3-cyano-prop-1-en-1-olate (*m*/*z* 81.9), 4-cyano-2-oxobutyrate (*m*/*z* 125.8), α-ketoglutaramate (*m*/*z* 144.1).

The aim of this study was to develop a method for the biocatalytic synthesis of preparative amounts of KGM (tens of grams), to minimize the contribution of side reactions, and to obtain the target product with a minimum content of impurities. The use of enzymes as catalysts (L-amino acid oxidase and catalase to remove the resulting hydrogen peroxide), as well as the optimization of the process conditions, made it possible to obtain a KGM preparation with a higher yield and chemical purity than those described in the literature. Biocatalytic synthesis of KGM (Figure 1) was performed using a highly concentrated (0.2 M) aqueous solution of the starting substrate L-glutamine. The kinetics of the formation of the target product and possible impurities was monitored using HPLC [15]. After the completion of the reaction, colorless crystals of the KGM sodium salt were isolated from the reaction mixture with a total yield of more than 75% (taking into account the stages of isolation and purification) and a chemical purity higher than 97% (according to HPLC data, Figure 2). Optimized conditions of HPLC analysis made it possible to identify impurities that are contained in the reaction mixture and may be present in the final product. According to the results of HPLC analysis, the 225 ± 6 mM KGM solution was obtained after carrying out preparative biocatalytic synthesis and concentration. Among the impurities present in the target product, the following were identified: 5-oxoproline at a concentration of about 1.2%, α-ketoglutarate at a concentration of about 0.03%, and residual amounts of the initial substrate L-glutamine at a concentration of less than 0.05%. MS/MS mass spectrum (Figure 3) confirms the structure of the synthesized KGM. In the MS/MS mode of detection, the corresponding identified fragments of the molecule are observed: 4-cyano-2-oxobutyrate and 3-cyano-prop-1-en-1-olate.

**Figure 4:**
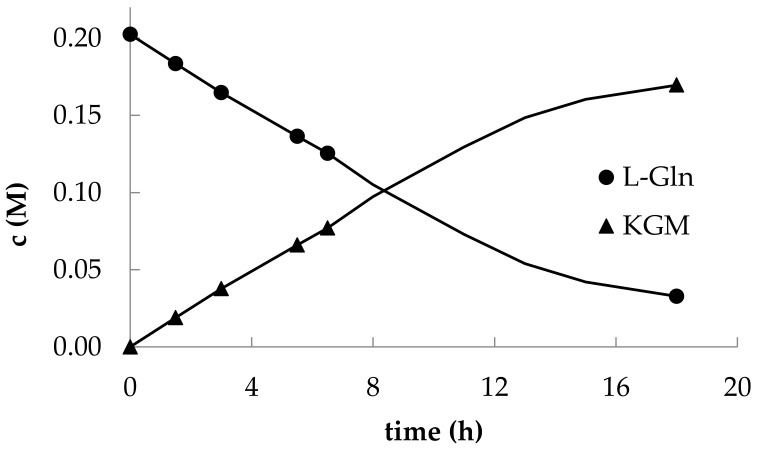
Representative kinetics of preparative production of α-ketoglutaramate by *C. adamanteus* L-amino acid oxidase-catalyzed reaction (37 °C, pH 7.4, 1 mg/mL) in the presence of catalase (0.03 mg/mL).

The optimized HPLC method for detecting the components of the reaction mixture made it possible to trace the kinetics of L-glutamine consumption and KGM accumulation (Figure 4).

It should be noted that in the early studies of the enzymatic synthesis of KGM, the initial ratio of L-glutamine and L-amino acid oxidase from *C. adamanteus* was indicated by weight as 5:2 [12,13]; however, the duration of the enzymatic synthesis was not indicated. Our experience shows that there is no need for such a high content of L-amino acid oxidase in the reaction mixture, and the amount of the enzyme can be reduced by an order of magnitude. At the same time, the level of conversion of L-glutamine reaches 90% in 18 h. It should also be noted that during the synthesis under these conditions, no significant inactivation of enzymes is observed, and the enzymes separated from the low molecular weight components of the reaction mixture (see Section 3.4. Isolation, purification and concentration of KGM) can be reused. The optimal choice of the concentration of the L-amino acid oxidase preparation from *C. adamanteus* is also important for the content of impurities in the reaction mixture. The authors of the aforementioned works indicated a significant content of α-ketoglutarate impurities in the final solution (~2%). We have found that the use of lower concentrations of L-amino acid oxidase in the reaction mixture can significantly reduce the rate of accumulation of the α-ketoglutarate byproduct. Perhaps this is due to the presence of traces of contaminating amidases in the snake venom lyophilizate that catalyze the hydrolysis of the amide group of KGM. Thus, as a result of preparative synthesis, we managed to obtain a KGM preparation with a trace content of α-ketoglutarate at a level of ~0.03%.

The KGM was purified from unreacted L-glutamine on a column with a Dowex cation exchange resin in the isocratic elution mode with distilled water. The method made it possible to reduce the L-glutamine content in the reaction mixture from 10% to ≤0.05% in relation to the KGM content, and also to remove ammonium and metal cations from the reaction mixture. Concentration and evaporation of a solution of the sodium salt of α-ketoglutaramic acid in a vacuum to dryness yielded colorless crystals of sodium KGM.

The structure of the synthesized KGM was confirmed by mass spectrometry; we also showed the quantitative conversion of the synthesized product to α-ketoglutarate by human ω-amidase (NIT2), for which KGM is a specific substrate. Using human ω-amidase (NIT2), the concentration of the target product in the synthesized samples was also determined. KGM was converted to α-ketoglutarate by ω-amidase, and the resulting α-ketoglutarate was analyzed by two methods using a reference material for calibration: by HPLC or spectrophotometrically at 430 nm after modification with 2,4-dinitrophenylhydrazine [13].

## 3. Materials and Methods

### 3.1. Reagents

All reagents were of the highest quality available. L-glutamine (pure, 99–101%, PanReac AppliChem, Barcelona, Spain); L-amino acid oxidase from *Crotalus adamanteus* lyophilized venom (Type I, dried venom, 0.3 unit/mg solid), catalase (from bovine liver lyophilized powder, 2000–5000 units/mg protein), and cation exchange resin (AmberChrom (formerly Dowex) 50WX4 hydrogen form) were obtained from Sigma–Aldrich Chemical, St. Louis, MO, USA.

### 3.2. Production of KGM

A weighed portion of 4.437 g of L-glutamine was dissolved in 150 mL of distilled water. The pH of the solution was adjusted to 7.4 with 1 M NaOH. The solution was transferred into a 5-L conical flask, 4.5 mg of catalase was added to remove the formed hydrogen peroxide, and a solution (~5 mL) containing 150 mg of L-amino acid oxidase from *C. adamanteus*, initially dialyzed against distilled water. The flask was loosely covered with aluminum foil with holes for aeration. The mixture was incubated in a thermostat shaker at 37 °C and stirring at 80 rpm. The reaction was carried out for 18 h.

### 3.3. Monitoring the Progress of the Biocatalytic Reaction

To track the progress of the reaction, 100 μL aliquots were taken from the reaction mixture and mixed with 900 μL of 67% aqueous acetonitrile to precipitate the protein. The mixture was centrifuged for 3 min at 10,000× *g*. An aliquot of 200 μL of centrifuged mixture was diluted with 800 μL of buffer solution (20 mM KH_2_PO_4_, pH 2.9) and analyzed by HPLC. The analysis conditions were as follows: AkzoNobel Kromasil Eternity-5-C18 column, 4.6 × 250 mm, mobile phase 1.5:98.5 (vol.%) Acetonitrile/buffer solution 20 mM KH_2_PO_4_, pH 2.9, flow rate 1 mL/min, UV-detection at 210 nm, volume of injected sample 20 μL, column temperature 25 °C. Retention times were: L-glutamine—2.59 min, KGM—3.22 min, α-ketoglutarate—3.55 min, 5-oxoproline—5.06 min.

### 3.4. Isolation, Purification, and Concentration of KGM

Upon completion of the KGM production, the reaction mixture was centrifuged at 12,000× *g* for 30 min. The supernatant was deproteinized by ultrafiltration using centrifugal filters (Amicon Ultra-15, cutoff 30 kDa, Merck Millipore, Ireland). Ultrafiltration was performed at 4 °C and 4000× *g* for 20–25 min. The resulting enzyme concentrate can be reused. The collected deproteinized solution was purified from unreacted L-glutamine by ion exchange on a column with Dowex cation exchange resin in the H^+^-form. Column parameters were: length 40 cm, diameter 20 mm, temperature control 4 °C. The eluent was passed through the column using a high-pressure pump at a rate of 6 mL/min. The ion exchange resin was preliminarily activated by sequential washing with 500 mL of bidistilled water, 100 mL of 1 M HCl, and 500 mL of bidistilled water until the aqueous eluate was neutral (pH 6.0–6.5). The deproteinized solution was loaded onto a Dowex activated resin column in H^+^-form. The mixture was eluted with bidistilled water at a rate of 6 mL/min. Fractions of the eluate were collected at a volume of 4 mL. Fractions containing α-ketoglutaramic acid were identified by pH using indicator paper (pH < 4). These fractions were pooled, and the pH was adjusted to 6.5 with 1 M NaOH. The solution of sodium KGM obtained after purification was concentrated by evaporation in a desiccator over anhydrous calcium chloride under reduced pressure (15–20 mm Hg) to a concentration of 200 mM. If necessary, under the same conditions, the solution can be evaporated to dryness until colorless crystals are obtained. The purity of sodium KGM according to HPLC analysis was 97.3%. The overall reaction yield, taking into account purification and concentration, was >75%.

## 4. Conclusions

In the course of the study, the method of the biocatalytic synthesis of the natural substrate of ω-amidase—KGM was optimized for obtaining preparative amounts of KGM in the form of a highly concentrated solution or colorless crystals of sodium salt with a total yield (taking into account the isolation and purification) of more than 75% and a chemical purity of more than 97%. The structure of the target compound was proved by the MS/MS method. The kinetics of the accumulation of KGM from L-glutamine catalyzed by L-amino acid oxidase from *C. adamanteus* in the presence of catalase was studied, and the importance of choosing the optimal concentration of the biocatalysts, as well as ensuring adequate aeration of the solution for a sufficient supply of air oxygen to the reaction mixture, was noted. The developed techniques can be used as prototypes for scaling up the processes of obtaining KGM and creating cost-effective biocatalytic technologies for its synthesis, isolation, and purification. The prospects and scale of industrial production of KGM will depend on the need for medical diagnostics in a method for determining the activity of human ω-amidase using KGM as a substrate. The synthesis of a compound is currently necessary to ensure ongoing research in order to clarify the prospects and scope of its further use.
